# Membrane-Associated Self-Assembly for Cellular Decision Making

**Published:** 2025-05-22

**Authors:** Samuel L. Foley, Margaret E. Johnson

**Affiliations:** T. C. Jenkins Department of Biophysics, Johns Hopkins University, Baltimore, Maryland, USA

## Abstract

Cellular decision-making based on information received from the external environment is frequently initiated by transmembrane receptors. These receptors are known to propagate such information by triggering a series of irreversible, energy-consuming reactions. While this active mechanism ensures switch-like responses, here we show how spontaneous molecular self-assembly on a two-dimensional substrate can similarly act as a tunable and robust switch for detecting receptors at physiological concentrations. This mechanism is much more sensitive than other passive mechanisms for receptor detection. We derive analytical expressions for critical receptor densities that switch on nucleation and growth of assemblies, in close agreement with equilibrium stochastic reaction-diffusion simulations. The theory developed provides testable predictions for how each component controls decision thresholds and magnitude of response.

Living systems receive diverse inputs from their surrounding environment, including neighboring cells, via a variety of molecules. These molecules, including essential nutrients[1], growth factors[2], and surface adhesion proteins[3], interact directly with transmembrane receptors bridging the outside of the cell to the cell interior[4]. To initiate the appropriate output response, the familiar form of information processing is for these local interactions to initiate a series of irreversible reactions triggered by enzymes, generating a selective and switch-like transition[5–8]. However, given the diversity of inputs and subsequent decisions (e.g. nutrient uptake, cell motion), can alternate molecular paradigms for receptor detection at a threshold density decouple sensing from energy expenditure while retaining the switch-like response? Endocytosis[9] and adhesion formation during development[10] and motility[11] are not classically considered “information processing” and yet each require detection of receptors at controlled times and places for proper function. Because each of these processes combine receptor binding with self-assembly, we propose that the assembly itself offers a reliable way to encode a decisive, localized response. We show here that equilibrium binding of monomers to receptors, which does not induce a sharp sensitivity to density changes, can be pushed to a switch-like response via self-assembly of monomers benefitting from a 2D search space.

The equilibrium self-assembly of monomers with a sufficient valency Z>1 and topology can transition sharply from free to assembled over small changes in concentration or binding free energy ΔG. This stems from reduction in the probability of monomer dissociation by $\text{exp}\left(\Delta G/k_{\text{B}}T\right)$, where $k_{\text{B}}T$ is the thermal energy, following the formation of a closed loop of monomers[12–14]. This cooperativity is emergent rather than explicitly built-in through allostery, which we exclude from the assembly[14–18]. We exploit this sensitivity to concentration changes by coupling lattice assembly to dimensional reduction on the surface[19] and subsequent receptor binding. Receptor detection thresholds thus emerge in our theory and simulation from reversible bimolecular interactions via the participation of discrete, multivalent monomers, without the chemical feedback common in pattern formation[20]. The role of the receptor is related to, but distinguishable from, membrane adsorption. By providing a second anchor to the surface following adsorption, receptors increase 2D monomer densities, promoting assembly that feeds back into reduced desorption via closed loops with the surface; hence there is an accumulation of effective cooperativity. Our framework makes the dependence on the volume-to-surface-area ratio ℓ≡𝒱/𝒜 transparent, as together with the changes to ΔG from 3D to 2D[21], these factors set the maximal gain in assembly yield from concentrating monomers in 2D[22]. Patterns and nucleation observed in models[20, 23] and experiments[24, 25] are strongly coupled to ℓ.

Achieving assembly-driven sensing and discrimination of receptor densities without activation or energy input requires proper tuning of interaction strengths relative to concentrations. Monomers should be native 3D species with concentrations well below the critical threshold for nucleation. Adsorption and confinement to the membrane[26] will move them towards the critical concentration[22], but to be effective sensors, they should remain below this concentration in the absence of receptors. We derive a free energy function that predicts the fraction of monomers in ordered lattices for a finite pool of monomers. Without receptors, our model resembles pre-wetting frameworks[27–29], albeit with the dense (lattice) phase restricted to 2D topologically. A primary difference (in addition to finite size) is that our free energy derives from specific discrete pairwise interactions, such that our parameters then map directly to experimental measureables like binding affinities; the continuum nature of pre-wetting[30, 31] renders physiologic relevance of phase-separating regimes hard to test. We derive an accurate closed-form approximation to the critical receptor threshold from our theory and quantitatively test it via stochastic simulations. Our results show that receptor sensing by 2D self-assembly is broadly achievable in living systems.

## Theory.—

Our goal is to predict the size of the assembled structure (if any) under the assumption that the solution concentration is too low to nucleate bulk assembly. To this end, we construct a minimal model of equilibrium self-assembly coupled to membrane localization and transmembrane receptor binding, deriving our surface free energy from a lattice model. Our assemblycompetent monomers A can bind to a single membrane site L and may also bind to a transmembrane receptor R once bound to the membrane[32]. The monomer valence Z specifies how many contacts monomers can make to other monomers. The key energetic parameters are the monomer-lipid association constant $K_{\text{a}}^{\text{AL}}$, the monomer-receptor association constant $K_{\text{a}}^{\text{AR}}$, and the monomer-monomer bond (free) energy ε. The procedure can be summarized as finding the simultaneous solution of the coupled equilibria that govern the state of the assembled structure on the membrane: monomer-membrane equilibrium, monomer-receptor equilibrium, and monomer-monomer surface equilibrium.

The first of these equilibria, for the binding of A and L to form AL, follows simply from the 3-dimensional bimolecular equilibrium constant,

(1) $$K_{\text{a}}^{\text{AL}}=\frac{c_{\text{AL}}}{c_{\text{A}}c_{\text{L}}}.$$

Throughout this work we will use $c_{\text{X}}\equiv N_{\text{X}}/𝒱$ to denote the volume concentration of species X, where $N_{\text{X}}$ is the copy number and 𝒱 is the system volume. For species which are restricted to move along the 2-dimensional membrane surface of area 𝒜, such as L and AL, $c_{\text{X}}$ refers to the effective 3-dimensional concentration, related to the 2-dimensional concentration $\rho_{\text{X}}\equiv N_{\text{X}}/𝒜$ by ℓ.

The second equilibrium equation is similar, but slightly modified due to the monomer-receptor reaction being confined to the membrane surface. We have[22]

(2) $$\gamma K_{\text{a}}^{\text{AR}}=\frac{c_{\text{RAL}}}{c_{\text{AL}}c_{\text{R}}},$$

where γ≡ℓ/h is a unitless *dimensionality factor*. The phenomenological dimensional reduction length scale h relates association constants in 2D and 3D, $K_{\text{a}}^{\text{X},3\text{D}}=hK_{\text{a}}^{\text{X},2\text{D}}$ [21, 33]. This parameter accounts for both entropic and enthalpic alterations to the binding equilibrium in 2D compared to 3D, and is typically on the order of the molecular length scale (*i.e.*, nm). In this work, unless otherwise noted, $K_{\text{a}}$ will refer to the 3D equilibrium constant, being the quantity most often measured experimentally[33].

**FIG. 1.**
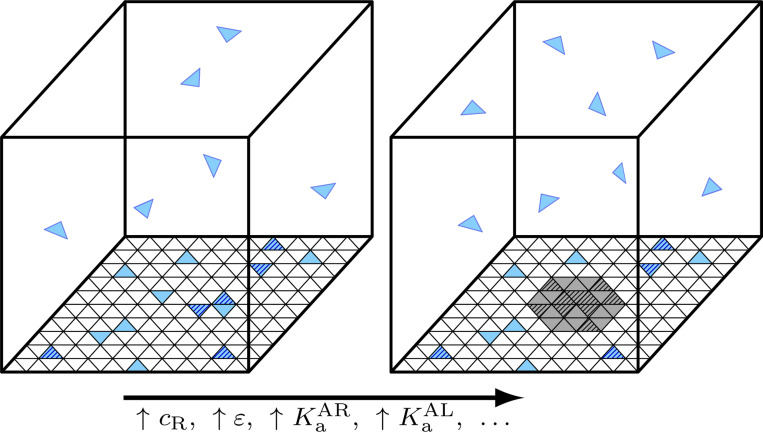
Schematic illustration of lattice model of receptorcoupled surface self-assembly. The membrane surface area (bottom) is discretized into a lattice Z=3 shown) which may be occupied by A monomers either in the free phase (blue) or coat phase (grey). Surface monomers bound to a receptor R are indicated with hashed shading.

The final equilibrium condition, monomer-monomer binding, is more complex due to its multivalency. We discretize the membrane surface into M total sites of area a (the monomer area), each of which may be occupied by a single lipid-bound monomer AL (Fig. 1). We simplify further by assuming that monomers are either free (not bound to any other monomer) or are part of the coat assembly. We denote the total number of monomers on the membrane by N and the number of free monomers by $N_{f}$. Defining the surface coverage fraction ϕ=N/M=Na/𝒜 and the free monomer fraction $\psi =N_{f}/N$, the approximate monomer entropy S, derived in the SI under the assumption of low total monomer coverage ϕ, is

(3) $$S=Nk_{\text{B}}\left[\phi \psi^{2}+(1-2\phi )\psi -\psi \text{ln}(\phi \psi )\right].$$

We pair this with a simple energy ansatz

(4) $$E=-\frac{1}{2}N_{c}Z\varepsilon k_{\text{B}}T+E_{b}\left(N_{c}\right),$$

in which $N_{c}=N-N_{f}$ is the number of monomers in the assembled coat. The unspecified function $E_{b}\left(N_{c}\right)$ is the energetic penalty of the coat boundary, where monomers are not fully saturated with bonds. The exact form of this energy contribution depends on Z and scales with $\sqrt{N_{c}}$. Putting together Equations (3) and (4), our free energy per surface monomer $f=(E-TS)/Nk_{\text{B}}T$ is

(5) $$f=\frac{1}{2}(\psi -1)Z\varepsilon +e_{b}(\phi ,\psi )-\phi \psi^{2}+(2\phi -1)\psi +\psi \text{ln}(\phi \psi ),$$

where $e_{b}=E_{b}/Nk_{\text{B}}T$. The equilibrium unassembled fraction ψ then follows from free energy minimization, requiring ∂f/∂ψ=0. The equilibrium value of ϕ follows from the coupling to Eqn. (1), which requires us to reexpress the concentrations of surface-bound A species in terms of ϕ and ψ (see SI). By doing so we can put all of our equilibrium equations into a consistent framework:

(6a) $$\left(c_{\text{A},\text{tot}}-\frac{\phi }{a\ell }\right)c_{\text{L}}K_{\text{a}}^{\text{AL}}=\frac{\phi \psi (1-\alpha )}{a\ell }$$

(6b) $$\left(c_{\text{R},\text{tot}}-\frac{\phi \alpha }{a\ell }\right)K_{\text{a}}^{\text{AR}}=\frac{\alpha }{\gamma \phi (1-\alpha )}$$

(6c) $$2\phi \psi -2\phi -\text{ln}(\phi \psi )=\frac{1}{2}Z\varepsilon +\frac{\partial e_{b}}{\partial \psi }$$

Eqns. (6a) and (6b) correspond to equations (1) and (2), respectively, while Eqn. (6c) is ∂f/∂ψ=0. We have introduced the new coordinate α corresponding to the fraction of membrane-bound monomers which are also bound to a receptor, which we assume is independent of whether the monomer is in the coat or free phase. Additionally, we treat the concentration $c_{\text{L}}$ as a constant under the reasonable assumption that lipid copies far exceed monomer copies [22].

**FIG. 2.**
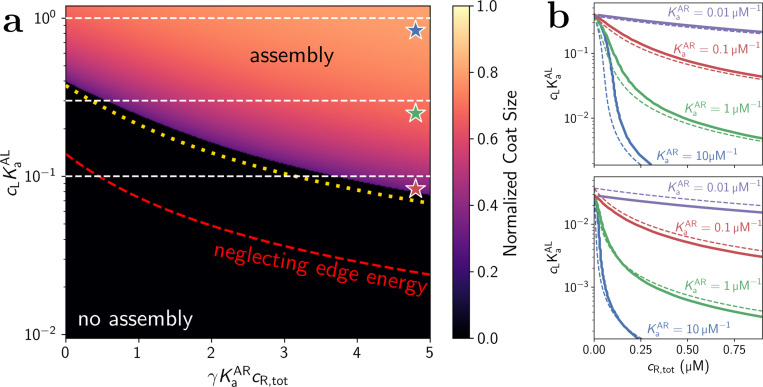
(**a**) Assembly phase diagram as adhesiveness (y-axis) and receptor binding (x-axis) are varied. $c_{\text{A},\text{tot}}=0.2\mu \text{M}$, $K_{\text{a}}^{\text{AR}}=0.1\mu {\text{M}}^{-1}$, Z=3, ε=5.1, h=10nm, and ℓ=1μm. Color shading (lighter is larger) indicates the overall fraction of monomers which have coalesced as found by numerically solving eqns. (6). The sharp transition indicates the nucleation boundary, with the dotted yellow curve being the analytical theory for this boundary given by the renormalized version of eqn. (7). The three dashed white lines and colored stars correspond to the series of stochastic simulation results shown in Fig. 3. (**b**) Top: Nucleation boundaries (equivalent to the sharp transition in left diagram) steepen vertically with stronger receptor binding strengths $K_{\text{a}}^{\text{AR}}$ as indicated, with other parameters being the same as in the **a**. Bottom: Same as above, but with ε=6.8. The overall shape of the contours change only at small R concentration, but the zero-R nucleation point shifts >10-fold lower on the adhesion axis. In all cases, exact numerical solutions are solid curves and analytical theory is dashed.

Fig. 2a plots an assembly “phase diagram” showing the predicted assembly size in the adhesiveness-receptor concentration plane $c_{\text{L}}K_{\text{a}}^{\text{AL}}$ *vs*. $c_{\text{R}}$) as found by numerically solving Eqns. (6). We find a contour along which the assembly size changes discontinuously, going from no assembly to nearly half of all monomers assembled on the membrane. We will refer to this as the assembly threshold or *decision threshold*[34]. The position and sharpness of the threshold varies with $K_{\text{a}}^{\text{AR}}$ and ε (Fig. 2b).

The system of equations (6) is transcendental and generally does not have an analytical solution in terms of elementary functions. However, if one assumes that the boundary energy $e_{b}$ is negligible compared to the bulk, it can be solved approximately via series expansion of the Lambert W function when exp(−Zε/2)≪1 (see SI), yielding a closed form for the critical receptor concentration $c_{\text{R}}^{*}$ above which assembly is stable:

(7) $$c_{\text{R}}^{*}=\frac{c_{\text{L}}K_{\text{a}}^{\text{AL}}}{a\ell }\frac{\left(a\ell c_{\text{A},\text{tot}}+\frac{ah}{c_{\text{L}}K_{\text{a}}^{\text{AL}}K_{\text{a}}^{\text{AR}}}-e^{-Z\varepsilon /2}\right)\left(e^{-Z\varepsilon /2}+\frac{e^{-Z\varepsilon /2}}{c_{\text{L}}K_{\text{a}}^{\text{AL}}}-a\ell c_{\text{A},\text{tot}}\right)}{a\ell c_{\text{A},\text{tot}}-e^{-Z\varepsilon /2}}.$$

This threshold is shown as the dashed red curve in Fig. 2a. As can be seen, the finite-size boundary energy $e_{b}$ generally cannot be neglected, as it significantly penalizes small coat sizes, resulting in a shift to the coat nucleation boundary. The numerically computed shading in Fig. 2 includes a finite size boundary term of the form

(8) $$E_{b}=\frac{1}{2}\sqrt{6N_{c}}\varepsilon k_{\text{B}}T,$$

corresponding to the lower bound of missing bond energy for Z=3 (see SI).The discontinuous assembly transition is shifted down in Fig. 2a compared to the extensive prediction (red dashed curve), indicating that for a fixed lipid binding strength, a higher concentration of receptors is required in order to initiate assembly due to the energetic penalization of small coats. However, the shape of the boundary is largely unchanged, and we can rescue equation (7) with the *ad hoc* energy rescaling $\varepsilon \to \left(1-2/\sqrt{3N_{\text{A}}}\right)\varepsilon$, where $N_{A}$ is the total number of A monomers. This represents an effective average weakening of the binding energy, by taking the edge energy at 50% assembly and distributing it evenly across all bonds. This modification is plotted as the dotted yellow curve in Fig. 2a, showing much closer agreement with the numerically determined assembly transition. Fig. 2b also shows the strong agreement between this analyticaly result and the numerically calculated exact solution.

## Bounds and robustness of sensing.—

Given Eqn. (7), we can characterize which systems are capable of sensing receptors and whether the threshold receptor concentration is robust to fluctuations in the membrane adhesiveness. Our results only depend on $c_{\text{L}}$ and $K_{\text{a}}^{\text{AL}}$ through the dimensionless product $y\equiv c_{\text{L}}K_{\text{a}}^{\text{AL}}$, which we call the adhesiveness. In the absence of R, equilibrium is determined solely through Eqns. (6a) and (6c). This simpler system tells us that if the adhesiveness is stronger than a critical strength

(9) $$c_{\text{L}}K_{\text{a}}^{\text{AL}}={\left(c_{\text{A},\text{tot}}a\ell e^{Z\varepsilon /2}-1\right)}^{-1},$$

then $c_{\text{R}}^{*}=0$, as no receptors are needed to nucleate assembly. Hence, if the monomer concentration, ℓ, or the strength ε are too high, receptors cannot be sensed. In Fig. 2a, this corresponds to the region above $c_{\text{L}}K_{\text{a}}^{\text{AL}}\approx 0.4$, where assembly is guaranteed regardless of $c_{\text{R}}$. Below this critical strength, the shape of the sensing decision boundary is directly tunable by $K_{\text{a}}^{\text{AR}}$ and h via Eqn. (7). As monomer-receptor interactions are strengthened by either increasing $K_{\text{a}}^{\text{AR}}$ or decreasing h, $c_{\text{R}}^{*}$ decreases.

This also changes the sensitivity of the threshold $c_{\text{R}}^{*}$ to variations in adhesiveness. The threshold is robust to variations in membrane adhesiveness when the decision boundary is sharp, or equivalently when $\text{d}c_{\text{R}}^{*}/\text{d}y$ (see Eqn. (S4)) is small. This occurs for strong interactions with receptors (high $K_{\text{a}}^{\text{AR}}$ or low h), whereas for weak interactions we see the receptor threshold changes rapidly with adhesiveness (Fig. 2b). In all cases, when the adhesiveness becomes very weak, the boundary line flattens out as expected, as eventually the receptors cannot rescue nucleation. Finally, either increasing ℓ or lowering the solution concentration of subunits $c_{\text{A},\text{tot}}$ both sharpen the decision threshold, supporting detection of low receptor concentrations, albeit also shifting the maximal membrane adhesion strength via Eqn. (9). Because $c_{\text{L}}$ is the 3D measure of recruiter concentration, we note the surface density $\rho_{L}$ must rise when ℓ increases and $c_{\text{L}}$ is constant[22].

## Stochastic simulations reproduce theory.—

**FIG. 3.**
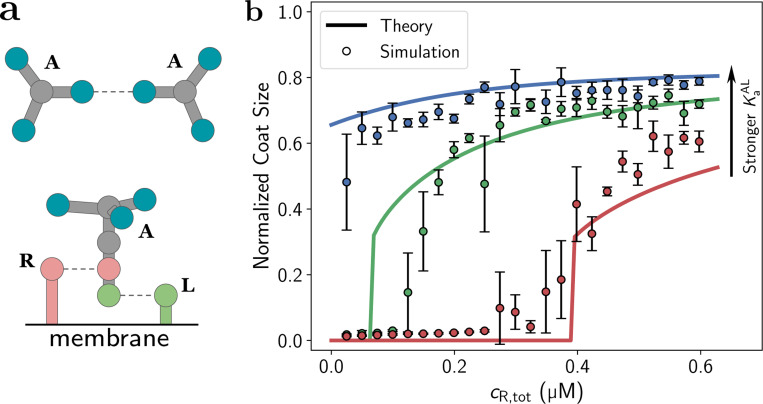
(**a**) Schematic diagram of the coarse-grained model used in reaction-diffusion (RD) simulations. The upper illustration shows a top-down view of monomer binding; the lower illustration gives a side-on view of A-R and A-L binding. (**b**) Equilibrium assembly size as a function of receptor concentration as determined from molecular RD simulations at 24 receptor concentrations. Error bars represent the standard error of the mean value from 4 simulations. The red, green, and blue colors correspond to $K_{\text{a}}^{\text{AL}}=3.3\times 10^{-3}\mu {\text{M}}^{-1}$, $0.01\mu {\text{M}}^{-1}$, and $0.033\mu {\text{M}}^{-1}$ respectively.

To validate the theory presented, we performed stochastic particle-based reaction-diffusion simulations using the NERDSS software package[35]. NERDSS generates stochastic trajectories of rigid-body components consistent with the Smoluchowski model of collision-based reactions[36, 37]. Binding and unbinding are parameterized by rates $k_{\text{on}}/k_{\text{off}}$ that directly constrain the equilibrium constants via $K_{\text{a}}=k_{\text{on}}/k_{\text{off}}$. Key inputs for the simulation are rigid molecule geometries with specified binding sites and binding partners, reaction rates, and copy numbers. We report all parameters in the SI. The model components mimic the lattice model used for the theory. Multivalent monomer subunits (A) each contain Z=3 sites to interact with other A monomers and assemble a hexagonal lattice, and they contain distinct sites to bind the lipids and receptors (Fig. 3a). Lipids and receptors have a single binding site. We include the one allosteric effect imposed in the theory: subunits bind receptors only after binding to the membrane.

By simulating each system to equilibrium, we quantify the size of the largest assembly as receptor densities increase, with direct comparison to the theoretical model of Eqns. (6) and Eqn. (8) (Fig. 3b). Simulations recapitulate the sharp transition in assembly for finite receptor densities at low adhesiveness, and the persistent nucleation without receptors for high adhesiveness. The parameter ε was determined by a single-parameter fit to all the data, with a best fit value of 5.1. The theory curves correspond to the horizontal dashed lines in Fig. 2. The overall agreement between theory and stochastic simulation is excellent.

## Biological and experimental relevance of parameter regimes.—

The parameter regimes used for simulations and theory closely match physiologic regimes for component concentrations[38, 39], binding affinities[39], and the ℓ of common eukaryotic cells[22]. To be concrete, we consider clathrin-mediated endocytosis, an essential process for internalizing receptors that is increasingly recognized as a key step during decision-making within cells[40]. The three components L, R and A map to the plasma membrane lipid PIP_2_, receptors such as Transferrin Receptor, and the clathrin trimer glued to its adaptor protein. Abundances and affinities used here were previously compiled in ref[23, 39], with details in SI.

Equilibrium in vitro studies show clathrin does not natively assemble in solution[41], but it does assemble following adaptor recruitment to lipid membranes, regardless of whether receptors are present[42, 43]. This failure to sense receptors is as predicted by our model when the adhesiveness of the membrane is sufficiently high. Indeed, the adhesiveness of in vitro membranes is at least 10-fold higher than in cells, typically from much higher PIP_2_ concentrations [42, 43]. Addition of receptor binding only enhances rather than triggers assembly[42]. An *in vitro* validation of our theory of a switch-like transition could be achieved by driving down PIP_2_ levels until membrane self-assembly is suppressed, followed by adding receptors. Performing this for varying levels of adhesiveness would directly probe the shape of Eqn. (7), the decision threshold.

## Equilibrium sensing is poor without assembly and dimensional reduction—

**FIG. 4.**
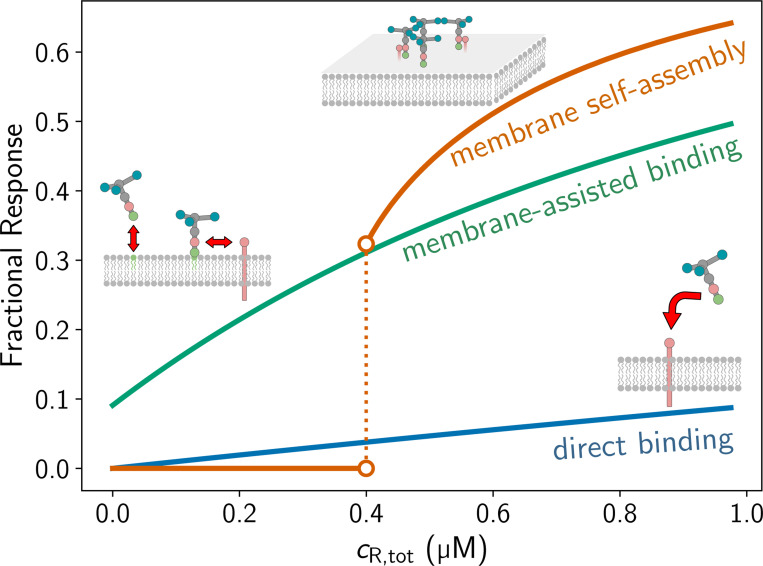
Comparison of different simple receptor “sensing” mechanisms. Direct binding is A binding directly to R, membrane-assisted binding is A first binding to a lipid L before binding to R. For these two cases, the quantity shown is the fraction of all A molecules which are on the membrane. For the case of membrane self-assembly, the fraction of all A molecules which are in the coat is plotted. $K_{\text{a}}^{\text{AR}}=0.1\mu {\text{M}}^{-1}$ across all examples and $c_{\text{L}}K_{\text{a}}^{\text{AL}}=0.1$.

Fig. 4 compares the sensitivity of self-assembly to that of simpler mechanisms relying on reversible bimolecular interactions, namely direct binding of monomers to target receptors and “membrane-assisted” binding, where the monomer first binds to the membrane and then the target receptor, but does not self-assemble. In both cases, the fraction of monomer bound to the membrane (the sensing signal strength) varies with the concentration of the target receptor, with membrane-assisted binding clearly being more sensitive. That the two-dimensional search is superior to direct binding (reliant on 3D diffusion) is not surprising, as previous work has shown that for our length- and time-scales of interest, two-dimensional diffusion is the energetically optimal mode of information transport[44]. Once 2D assembly is added to the sensing mechanism, the response is both larger in amplitude and exhibits a signal jump at a characteristic threshold, two distinct advantages over the simpler schemes.

## Discussion—

While our model does not feature self-clustering receptors, the analysis presented carries to such cases, as this corresponds to a *local* increase in receptor density. Such spatial control can be exploited to trigger assembly nucleation at specific locations and with improved cooperativity[45], either through direct receptor-receptor interactions[4], or through preferential partitioning into a particular membrane phase [27], e.g., ${\text{L}}_{\text{o}}/{\text{L}}_{\text{d}}$ phase separation or raft-like domains[46, 47]. Meso-scale mechanical properties such as membrane rigidity and tension can also couple to the decision threshold, as the membrane remodeling energy must be provided by the assembling protein machinery. The association free energies therefore must be able to offset this work barrier to be successful in absence of active energy-consuming processes[48].

An advantage of membrane-associated self-assembly as a sensing mechanism is to naturally “package” the molecules it senses, whether receptors[32] or RNA[49]. Moreover, self-assembly is responsive to increases in receptor densities beyond the target threshold (Fig. 4). Abrupt increases in external stimuli (such as Calcium) would be rapidly (i.e. seconds[50]) followed by increasing assembly, as seen in excitable cells[51]. Maintaining homeostasis in cells, including in cell crawling and endocytosis, requires active energy input to recycle receptors on/off the membrane[9], but our model demonstrates that this necessary activity can be coupled to downhill self-assembly to relax to the homeostatic “set point” for receptor density. The disadvantage of this mechanism is that it is not well-suited for sensitive detection of ultra-dilute components like a single receptor that is possible via signal transduction[52, 53], as that would not be sufficient to promote assembly. Close to the decision threshold, there are large fluctuations in assembly due the sharpness of the transition, and living systems may operate near this threshold: For endocytosis, about 50% of initiated assembly events are observed to be unsuccessful[54].

By expanding beyond the three distinct components incorporated here, we expect our model to reproduce the phenomenology of ‘multifarious’ self-assembly models, where diverse subunits facilitate tunable, separable condensates[16], optimal assembly kinetics[12, 15], and programmable nucleation and classification[18]. With dozens of distinct, multivalent components the norm in eukaryotic biology in both structured and disordered assemblies[55, 56], these systems could be tuned to establish multiple detection limits for distinct receptors by exploiting the sensitivity of nucleation to variations in concentration (or affinities), demonstrating nucleation-driven pattern recognition[17, 18] in living systems.

## Supplementary Material

Supplement 1
