# Supplementary material for: Membrane-Associated Self-Assembly for Cellular Decision Making
Source: ArXiv. 2025 May 22:arXiv:2505.17290v1. Preprint. [Version 1] (PMC12136478)

Supplementary Information for:
Membrane-Associated Self-Assembly for Cellular Decision Making

Samuel L. Foley and Margaret E. Johnson

1 Surface Free Energy

Consider a 2-dimensional lattice with M total sites that can be occupied by self-assembly monomers A. There are N of these A in total on the surface. We will work in the dilute limit where we assume $N \ll M$. We will denote the number of free individual A monomers as N_f, so that $N_f \leq N$. The remaining $N - N_f$ monomers are assumed to reside in a single coat. Here is an example lattice configuration:

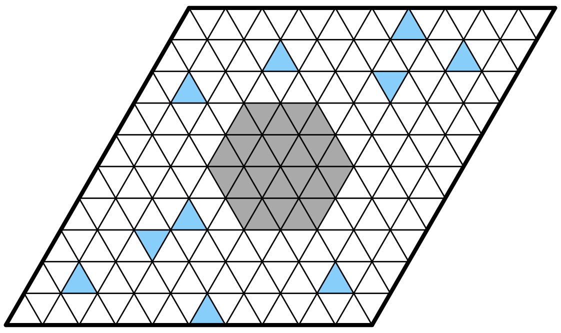

In this example, $M = 200$, $N = 34$, $N_f = 10$. The number of ways to arrange the N_f free monomers is

$$\binom{M-(N-N_f)}{N_f} = \frac{(M-(N-N_f))!}{N_f!(M-(N-N_f)-N_f)!} = \frac{(M-N+N_f)!}{N_f!(M-N)!}$$

The $O(M)$ positions for the coat represent a small contribution compared to this entropy, so we will ignore the coat entropy entirely. Thus we take as our entropy

$$S = k_\mathrm{B} \ln\left[\frac{(M-N+N_f)!}{N_f!(M-N)!}\right].$$

Unpacking this using rules of logarithms and applying Stirling's approximation yields

$$\frac{S}{k_\mathrm{B}} \approx (M-N+N_f)\ln M + (M-N+N_f)\ln\left(1 - \frac{N-N_f}{M}\right) - N_f \ln N_f - (M-N)\ln M - (M-N)\ln\left(1-\frac{N}{M}\right)$$

Linearizing $\ln(1+x) \approx x$ for small x and simplifying, we have

$$\frac{S}{k_\mathrm{B}} \approx N_f \ln M + (M-N)\frac{N_f}{M} + N_f\frac{N_f - N}{M} - N_f \ln N_f.$$

We now introduce the following coordinates:

$$\phi = \frac{N}{M} \qquad \psi = \frac{N_f}{N}.$$

ϕ is the overall surface coverage and ψ is the fraction of free monomers. In the example shown above, $\phi \approx 0.17$ and $\psi \approx 0.29$. With these, our entropy becomes Eqn. (3) of the main text:

$$\frac{S}{Nk_\mathrm{B}} = \phi\psi^2 + (1-2\phi)\psi - \psi\ln(\phi\psi). \tag{S1}$$

We make the simplest possible assumption for the energy: each monomer in the coat has Z neighbors ($Z = 3$ in our example shown above, as for a clathrin-like assembly), and we will attribute $-\varepsilon k_\mathrm{B}T$ to each bond, meaning

$$E = -\frac{1}{2}(N-N_f)Z\varepsilon\, k_\mathrm{B}T.$$

Thus, our free energy is

$$f = \frac{E - TS}{Nk_\mathrm{B}T} \approx \frac{1}{2}(\psi - 1)Z\varepsilon - \phi\psi^2 + (2\phi - 1)\psi + \psi\ln(\phi\psi).$$

Fig. S1a plots $f(\psi)$ for $\varepsilon = 2$, $Z = 3$ and a few values of overall surface coverage ϕ. For this bond energy, coat assembly begins at $\phi_c \approx 0.05$, or 5% surface coverage, from which point the coat grows with increasing ϕ. Fig. S1b plots the fraction of monomers within the coat, $1 - \psi$, as a function of ϕ, as found by minimizing the free energy. We will now derive analytical expressions for the critical coverage ϕ_c at which coat nucleation begins, as well as for the equilibrium fraction of free monomers, ψ_{eq}.

$$\frac{\partial f}{\partial \psi} = \frac{1}{2}Z\varepsilon - 2\phi\psi + 2\phi + \ln(\phi\psi)$$

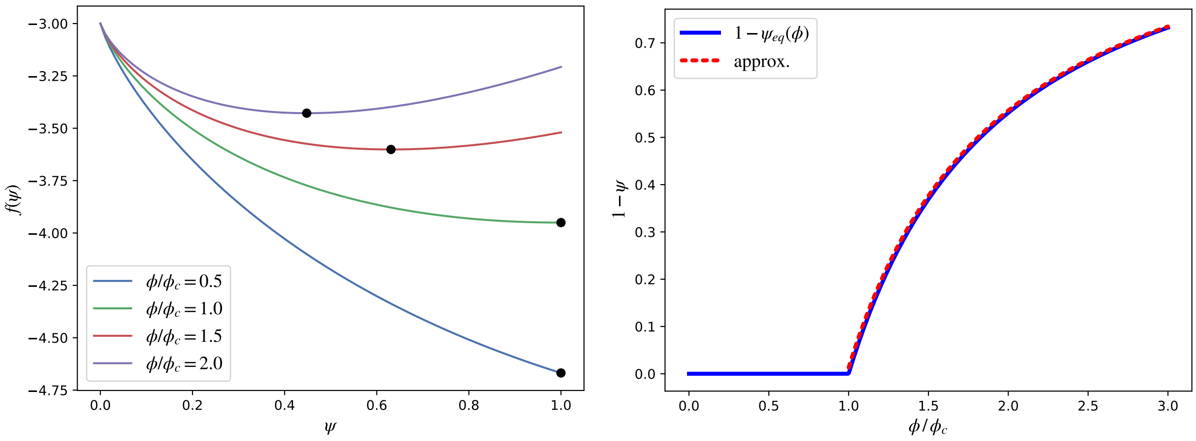

Figure S1: (a) Plots of eqn. (1) for various values of surface coverage ϕ with $\varepsilon = 2$, $Z = 3$ ($\phi_c \approx 0.05$). The black point on each curve indicates the free energy minimum. (b) Plot of equilibrium fraction of monomers in the assembled coat, $1 - \psi_{eq}$. Dashed red shows the approximation that avoids using special functions.

The equilibrium value of ψ is found by setting this equal to zero, with the restriction that $0 \leq \psi \leq 1$. We can find ϕ_c by inserting $\psi = 1$ and solving for the ϕ that makes the above zero,

$$\frac{1}{2}Z\varepsilon + \ln(\phi_c) = 0 \quad \rightarrow \quad \phi_c = e^{-Z\varepsilon/2}$$

For $\phi \leq \phi_c$, $\psi_{eq} = 1$. For $\phi > \phi_c$, we have to minimize f, meaning solving for the roots $\psi(\phi)$ of $\partial f/\partial\psi$, which is transcendental, having solution

$$\psi(\phi) = -\frac{1}{2\phi}W_0\left(-2e^{-2\phi - Z\varepsilon/2}\right), \qquad \phi > \phi_c\,.$$

Here $W_k(x)$ is the kth branch of the Lambert W function, the inverse of $f(W) = We^W$, with $W_0(x)$ being the "principal branch." This solution was used to plot the blue curve in Fig. S1b. A very good approximation is acquired by series expanding $W_0(x)$ about $x = 0$,

$$W_0(x) \approx x - x^2$$

$$\implies \psi(\phi) \approx \frac{e^{-2\phi - Z\varepsilon/2}}{\phi}\left(1 + 2e^{-2\phi - Z\varepsilon/2}\right), \qquad \phi > \phi_c.$$

This was used to plot the red dotted curve in Fig. S1b, which is scarcely distinguishable from the exact result. As ε increases this approximation becomes better. For sufficiently small ϕ_c one can also ignore the second term in the expansion: at $\varepsilon = 2$ (the case plotted above), it accounts for roughly 9% of the value, while at $\varepsilon = 4$ it has decreased to less than half a percent. For the model simulated in the main text, where $\varepsilon \approx 5.1$, it is entirely negligible. We then take

$$\psi_{eq}(\phi) = \begin{cases} 1 & \phi \leq \phi_c \\ \dfrac{e^{-2\phi - Z\varepsilon/2}}{\phi} & \phi > \phi_c \end{cases}$$

2 Membrane Binding Equilibrium

We will denote the surface area to volume ratio as $\ell = V/A$, and each monomer takes up a surface area a_0. The relevant concentration variables in terms of our free energy coordinates ϕ and ψ are

$$M = \frac{A}{a_0} \implies c_{\text{A,mem}} = \frac{\phi M}{V} = \frac{\phi}{a_0\ell} \implies c_{\text{A}} = c_{\text{A,tot}} - \frac{\phi}{a_0\ell} \qquad c_{\text{AL}} = \frac{\phi\psi}{a_0\ell}$$

Solving for ϕ from the binding equilibrium given by K_{a}^{AL} gives

$$K_{\text{a}}^{\text{AL}} = \frac{c_{\text{AL}}}{c_{\text{A}}c_{\text{L}}} = \frac{\phi\psi/a_0\ell}{\left(c_{\text{A,tot}} - \frac{\phi}{a_0\ell}\right)c_{\text{L}}} \qquad \rightarrow \qquad a_0\ell c_{\text{A,tot}} - \phi\left[1 + \frac{\psi(\phi)}{c_{\text{L}}K_{\text{a}}^{\text{AL}}}\right] = 0$$

This yields the self-consistent value for the equilibrium area coverage ϕ_{eq} as a function of total monomer concentration $c_{\text{A,tot}}$ taking into account monomers binding and un-binding from the surface. Taking the approximated form of ψ_{eq}, eqn. (1), we can once again solve in terms of the Lambert W function:

$$\phi_{eq}^{+}(c_{\text{A,tot}}) \approx a_0\ell c_{\text{A,tot}} + \frac{1}{2}W_0\left(-\frac{2\phi_c e^{-2a_0\ell c_{\text{A,tot}}}}{c_{\text{L}}K_{\text{a}}^{\text{AL}}}\right)$$

Recalling that $\psi_{eq}(\phi)$ is a piecewise function, and that this solution for ϕ_{eq} was calculated with the piece that is only valid for $\phi > \phi_c$, the other piece, where $\psi_{eq} = 1$, gives

$$\phi_{eq}^{-}(c_{\text{A,tot}}) = c_{\text{L}}K_{\text{a}}^{\text{AL}}\frac{a_0\ell c_{\text{A,tot}}}{1 + c_{\text{L}}K_{\text{a}}^{\text{AL}}}.$$

The crossover between the two happens at the point where ϕ_{eq} reaches ϕ_c, which allows us to define the critical total bulk concentration c^*_{A} required for coat nucleation:

$$c^*_{\mathrm{A}} = \frac{\phi_c}{a_0\ell}\left(1 + \frac{1}{c_{\mathrm{L}}K_{\mathrm{a}}^{\mathrm{AL}}}\right) = \frac{e^{-Z\varepsilon/2}}{a_0\ell}\left(1 + \frac{1}{c_{\mathrm{L}}K_{\mathrm{a}}^{\mathrm{AL}}}\right).$$

And with this, we can write

$$\phi_{eq}(c_{\mathrm{A,tot}}) = \begin{cases} \phi^-_{eq}(c_{\mathrm{A,tot}}) & c_{\mathrm{A,tot}} \leq c^*_{\mathrm{A}} \\ \phi^+_{eq}(c_{\mathrm{A,tot}}) & c_{\mathrm{A,tot}} > c^*_{\mathrm{A}} \end{cases}$$

We can also once again write an approximate solution,

$$\phi^+_{eq} \approx a_0\ell c_{\mathrm{A,tot}} - \frac{\phi_c e^{-2a_0\ell c_{\mathrm{A,tot}}}}{c_{\mathrm{L}}K_{\mathrm{d}}^{\mathrm{AL}}}.$$

The fraction of all A monomers which have coalesced is

$$\phi M \times (1-\psi)/(c_{\mathrm{A,tot}}\mathcal{V}) = \frac{\phi(1-\psi)}{a\ell c_{\mathrm{A,tot}}}$$

3 Receptor Equilibrium

For this step we will introduce the approximation that the probability for an A to be bound to a receptor R is independent of whether or not it is in the coat. We define the fraction of membrane-bound A which are bound to R as α. Then we can say

$$c_{\mathrm{AL}} = \frac{\phi\psi}{a_0\ell}(1-\alpha),$$

and follow the same steps as above. We determine α from $K_{\mathrm{a}}^{\mathrm{AR}}$ (Eqn. (2) in the main text),

$$\gamma K_{\mathrm{a}}^{\mathrm{AR}} = \frac{\frac{\phi\alpha}{a_0\ell}}{\frac{\phi(1-\alpha)}{a_0\ell}\left(c_{\mathrm{R,tot}} - \frac{\phi\alpha}{a_0\ell}\right)} = \frac{\alpha a_0\ell}{(1-\alpha)(a_0\ell c_{\mathrm{R,tot}} - \phi\alpha)}$$

$$\implies \alpha(\phi, c_{\mathrm{R,tot}}) = \frac{1}{2\phi}\left[\phi + a_0\ell c_{\mathrm{R,tot}} + \frac{a_0\ell}{\gamma K_{\mathrm{a}}^{\mathrm{AR}}} - \sqrt{\left(\phi + a_0\ell c_{\mathrm{R,tot}} + \frac{a_0\ell}{\gamma K_{\mathrm{a}}^{\mathrm{AR}}}\right)^2 - 4\phi a_0\ell c_{\mathrm{R,tot}}}\right]$$

The net result is that now we determine ϕ_{eq} by finding the roots of

$$a_0\ell c_{\mathrm{A,tot}} - \phi\left[1 + \frac{\psi(\phi)}{c_{\mathrm{L}}K_{\mathrm{a}}^{\mathrm{AL}}}(1 - \alpha(\phi, c_{\mathrm{R,tot}}))\right] = 0. \tag{S2}$$

From this we can find nucleation boundaries for different parameter combinations. One of the simpler results is for c^*_{A} considered as a function of total receptor concentration $c_{\mathrm{R,tot}}$, which we can solve for in the same way as before: set $\psi = 1$ and $\phi = \phi_c$ and solve,

$$c^*_{\mathrm{A}}(c_{\mathrm{R,tot}}) = \frac{\phi_c}{a_0\ell}\left[1 + \frac{1 - \alpha(\phi_c, c_{\mathrm{R,tot}})}{c_{\mathrm{L}}K_{\mathrm{d}}^{\mathrm{AL}}}\right]$$

$$= \frac{e^{-Z\varepsilon/2}}{a_0\ell}\left[1 + \frac{1}{c_{\mathrm{L}}K_{\mathrm{a}}^{\mathrm{AL}}}\left(1 - \frac{e^{Z\varepsilon/2}}{2}a_0\ell\left[\frac{e^{-Z\varepsilon/2}}{a_0\ell} + c_{\mathrm{R,tot}} + \frac{1}{\gamma K_{\mathrm{a}}^{\mathrm{AR}}} - \sqrt{\left(\frac{e^{-Z\varepsilon/2}}{a_0\ell} + c_{\mathrm{R,tot}} + \frac{1}{\gamma K_{\mathrm{a}}^{\mathrm{AR}}}\right)^2 - \frac{4e^{-Z\varepsilon/2}c_{\mathrm{R,tot}}}{a_0\ell}}\right]\right)\right].$$

The only difference from the no-receptor case is the factor of $(1-\alpha)$ inside the brackets. This will show us how the necessary adaptor amount $c_{\mathrm{A,tot}}$ for coat nucleation changes as we vary the receptor abundance $c_{\mathrm{R,tot}}$.

More interesting is to instead solve for $c_{\mathrm{R,tot}}$ to acquire a direct formula for the receptor-triggered nucleation threshold for a given monomer concentration. The result in this case is

$$c^*_{\mathrm{R}} = \frac{c_{\mathrm{L}}K_{\mathrm{a}}^{\mathrm{AL}}}{a\ell}\frac{\left(a\ell c_{\mathrm{A,tot}} + \frac{ah}{c_{\mathrm{L}}K_{\mathrm{a}}^{\mathrm{AL}}K_{\mathrm{a}}^{\mathrm{AR}}} - e^{-Z\varepsilon/2}\right)\left(e^{-Z\varepsilon/2} + \frac{e^{-Z\varepsilon/2}}{c_{\mathrm{L}}K_{\mathrm{a}}^{\mathrm{AL}}} - a\ell c_{\mathrm{A,tot}}\right)}{a\ell c_{\mathrm{A,tot}} - e^{-Z\varepsilon/2}}$$

which is Eqn. (7) of the main text. To gain quantitative insight into the robustness and sensitivity of this threshold, we can examine its derivtive with respect to the adhesiveness $y \equiv c_{\mathrm{L}}K_{\mathrm{a}}^{\mathrm{AL}}$:

$$\frac{\mathrm{d}c^*_{\mathrm{R}}}{\mathrm{d}y} = \frac{e^{-Z\varepsilon/2} - a\ell c_{\mathrm{A,tot}}}{al} + \frac{e^{-Z\varepsilon/2}}{y^2\gamma K_{\mathrm{a}}^{\mathrm{AR}}(e^{-Z\varepsilon/2} - a\ell c_{\mathrm{A,tot}})} \tag{S3}$$

Evaluated at the critical adhesiveness given by Eqn. (9) in the main text, this becomes

$$\frac{\mathrm{d}c^*_{\mathrm{R}}}{\mathrm{d}y} = -c_{\mathrm{A,tot}} + \frac{e^{-Z\varepsilon/2}}{al} - \frac{ahc_{\mathrm{A,tot}}}{K_{\mathrm{a}}^{\mathrm{AR}}}e^{+Z\varepsilon/2} + \frac{1}{\gamma K_{\mathrm{a}}^{\mathrm{AR}}} \tag{S4}$$

This derivative is negative, and its magnitude decreases with increasing $K_{\mathrm{a}}^{\mathrm{AR}}$ or ℓ, but increases with increasing h or c_{A}.

4 Finite-Size Edge Energy

In our free energy, eqn. (1), we assume a contribution of $-Z\varepsilon$ for every monomer in the assembly. However, those monomers which reside on the edge of the coat will have fewer than Z bonded neighbors. This constitutes a free energy penalty that is proportional to the perimeter. The exact details of this edge free energy penalty depend on the coat geometry, i.e., the form penalty term is Z-dependent.

We will treat the $Z = 3$ case considered in the main text using the simplest approximation. Let N_c be the number of monomers in the coat. For "closed shell" configurations like in the in the example pictured at the start ($N_c = 6, 24, 54, 96, ...$), the number of monomers in the outermost layer is $2\sqrt{6N_c} - 6$. The number of these which constitute the "edge", meaning they are missing exactly one bond, is $\sqrt{6N_c}$. Thus the penalty to the free energy per monomer on the membrane is

$$\Delta f = \frac{\sqrt{6N_c}}{2N}\varepsilon.$$

Extrapolating this to all N_c, not just the closed-shell values, gives us a lower-bound approximation for the edge energy. Recognizing that $N_c = N(1-\psi)$ and $N = \mathcal{A}\phi/a$, this becomes

$$\Delta f = \frac{1}{2}\varepsilon\sqrt{\frac{6a(1-\psi)}{\mathcal{A}\phi}}. \tag{S5}$$

5 Parameter regimes that are physiologically relevant to clathrin-mediated endocytosis

Parameters were previously collected for abundances and affinities of component for endocytosis, but we expand here. Receptor concentrations can vary widely, but the span we use here (Fig 3) encompasses Transferrin receptor from distinct cell types (from $36\,\mu\text{m}^{-3}$ to roughly $1000\,\mu\text{m}^{-3}$), and integrin subtypes that reach 10-fold lower still. Because the abundant clathrin trimers ($\sim 0.6\,\mu\text{M}$) need adaptors to bind the membrane, we limit the A copies to the abundance of the central adaptor protein AP2 at $0.2\,\mu\text{M}$. The K_a^AA value we used is about 4-fold weaker than estimated clathrin-clathrin binding, but this is offset by additional negative cooperativity in clathrin assembly stability needed to reproduce *in vitro* assembly kinetics on membranes[**guo2022large**]. Finally, K_a^AL should capture the strength of adaptor-lipid binding ($\sim 0.1\,\mu\text{M}^{-1}$) and clathrin-adaptor binding ($\sim 0.04\,\mu\text{M}^{-1}$) for an effective lipid binding strength in the $K_\text{a}^\text{AL} \sim 10^{-3}\,\mu\text{M}^{-1}$ range.

6 Simulation Parameters

Time step: $\Delta t = 1\,\mu\text{s}$

Total iterations: $7.2 \times 10^8 \implies t_\text{sim} = 720\,\text{s}$

Box geometry: $1\,\mu\text{m} \times 1\,\mu\text{m} \times 1\,\mu\text{m}$

Fixed initial species counts:

$N_\text{L} = 18{,}000$

$N_\text{A} = 120$

Simulation series: Three sets of simulations were performed, each varying the receptor copy number:

$N_\text{R} = 15$ to 360 in increments of 15 (24 values total)

Replicas: 4 independent simulations per N_R value

Macroscopic rate constants:

$$k_\text{on}^\text{AL} = 0.3\,\mu\text{M}^{-1}\text{s}^{-1} \qquad k_\text{off}^\text{AL} = K_\text{d}^\text{AL} \times k_\text{on}^\text{AL} \qquad K_\text{d}^\text{AL} = \{30,\ 100,\ 300\}\,\mu\text{M}$$

$$k_\text{on}^\text{AR} = 1\,\mu\text{M}^{-1}\text{s}^{-1} \qquad k_\text{off}^\text{AR} = 10\,\text{s}^{-1}$$

$$k_\text{on}^\text{AA} = 0.2\,\mu\text{M}^{-1}\text{s}^{-1} \qquad k_\text{off}^\text{AA} = 10\,\text{s}^{-1}$$

Diffusion constants: Each species has an isotropic translational and rotational diffusion constant:

$$D_\text{A}^\text{trans} = 25\,\text{nm}^2\,\text{s}^{-1} \qquad D_\text{L}^\text{trans} = 1\,\text{nm}^2\,\text{s}^{-1} \qquad D_\text{R}^\text{trans} = 1\,\text{nm}^2\,\text{s}^{-1}$$

$$D_\text{A}^\text{rot} = 0.5\,\text{rad}^2\,\mu\text{s}^{-1} \qquad D_\text{L}^\text{rot} = 0 \qquad D_\text{R}^\text{rot} = 0$$

L and R have no rotational diffusion in simulation because their CG models are rotationally symmetric with respect to their only free rotation axis (only one free rotation axis due to confinement to the 2D membrane surface).

Simulation CG model geometry (not to scale):

A bound to L and R:

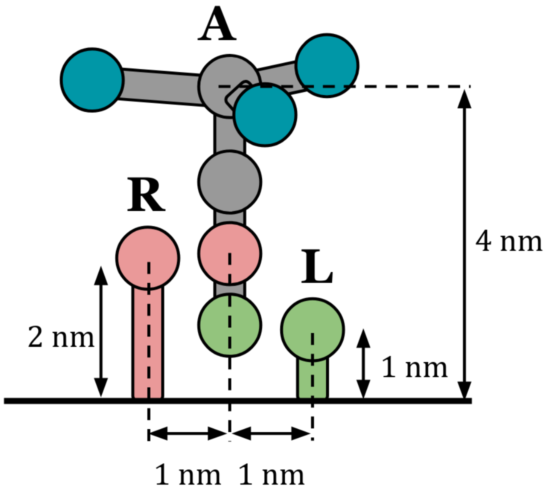

Top-down A-A binding:

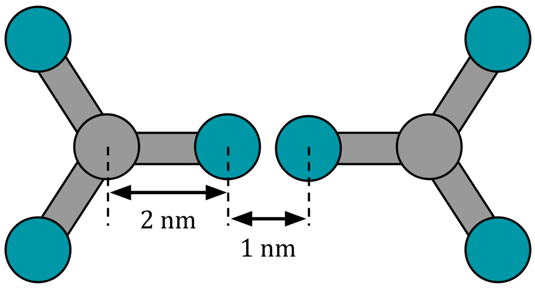

Supplement: Supplement 1 [file NIHPP2505.17290v1-supplement-1.pdf]
